# Adjuvant sorafenib for hepatocellular carcinoma after radiofrequency ablation versus radiofrequency ablation: analysis of its efficacy and safety

**DOI:** 10.3389/fonc.2024.1383312

**Published:** 2024-12-04

**Authors:** Wang Junxiao, Liu Rui, Wen Zhenyu, Sang Zejie, Yang Xiang, Ding Mingchao, Xie Hui

**Affiliations:** ^1^ Aerospace Medical Center, Aerospace Center Hospital, Beijing, China; ^2^ Senior Department of Oncology, Fifth Medical Center of Chinese PLA General Hospital, Beijing, China; ^3^ Department of Interventional Vascular Surgery, Aerospace Center Hospital, Beijing, China

**Keywords:** hepatocellular carcinoma, radiofrequency ablation, sorafenib, computed tomography guided, efficacy and safety

## Abstract

**Objectives:**

For the treatment of early hepatocellular carcinoma, we compared the efficacy and safety of radiofrequency ablation (RFA) alone and radiofrequency ablation combined with sorafenib (RFA+Sor).

**Methods:**

A total of 164 patients with early HCC were included in the study. There were 87 patients who underwent RFA alone, and 77 patients who underwent RFA+Sor treatment. Overall survival (OS) was the primary endpoint of the study, and recurrence-free survival (RFS) and safety were the secondary endpoints.

**Results:**

According to the RFA group, the RFS rates were 74.7%, 29.9%, and 11.5% at 1, 2, and 3 years, whereas in the RFA+Sor group, the RFS rates were 72.7%, 19.5%, and 11.7% at 1, 2, and 3 years (*P*>0.05). RFA and RFA+Sor groups had median OS of 35.0 and 41.0 months, respectively (*P*>0.05). For the RFA and RFA+Sor groups, the median RFS was 17.0 and 16.0 months, respectively (*P*>0.05). Based on the univariate regression analysis, there was no statistically significant difference between the subgroups (*P*>0.05). Skin rashes only occurred in the RFA+Sor group, and other adverse effects were not significantly different between the two groups (*P*>0.05).

**Conclusions:**

Treatment with RFA+Sor treatment did not result in a longer OS than treatment with only RFA, however, the adverse effects of adjuvant Sorafenib were acceptable.

## Introduction

Primary liver cancer is the sixth most common malignant tumor in the world, and its incidence has increased in recent years; it has the fourth highest mortality rate in the world. More than half of the world’s primary liver cancer cases occur in China, with hepatocellular carcinoma (HCC) accounting for 90% (1, 2). In most cases, HCC is associated with liver cirrhosis, such as chronic hepatitis B virus infection, chronic hepatitis C virus infection, or non-alcoholic steatohepatitis (3, 4).

Patients diagnosed at an early stage of the disease may be treated with liver resection, transplantation, and local ablation as radical treatments; according to a study (3–6), patients who underwent liver resection had a 5-year survival rate of 60-80%, while patients who underwent local ablation had a survival rate of 40-70%. The tumor recurrence rate, however, is high, which affects the overall survival of these patients. A study reported that the 3-year tumor recurrence rate was approximately 50% and 70%, respectively (3–9). In conclusion, the long-term prognosis of HCC remains unsatisfactory after ablation, and preventing tumor recurrence or prolonging survival through adjuvant treatment remains a significant medical challenge.

As HCC is a highly heterogeneous tumor, and most patients have varying degrees of liver disease, adjuvant treatment presents a significant challenge. A standard adjuvant treatment for HCC does not exist at present because no adjuvant treatment has been shown to be effective in randomized studies after conventional treatment (4, 10–12). In spite of the fact that interferon is the most commonly used adjuvant treatment for HCC patients who undergo radical treatment, the evidence is limited and based on studies with small sample sizes, different patients, and different treatment durations (13). In a multicenter randomized clinical trial, Huaier granules proved to be an effective treatment after curative liver resection, but not after local ablation (14). There has been no convincing evidence that other adjuvant treatments after radical treatment, such as vitamin K2 and systemic chemotherapy, are effective or safe (15, 16).

The oral tyrosine kinase inhibitor sorafenib is approved for the treatment of unresectable HCC based on two phase 3 randomized trials (17, 18), and it is the most commonly prescribed treatment for advanced HCC (3, 11). Recurrence of tumors is associated with minimal residual disease (6, 19). Thus, sorafenib is a rational adjuvant treatment for HCC patients who undergo radiofrequency ablation (RFA) because it inhibits angiogenesis and tumor cell proliferation (20). It is unclear whether sorafenib is effective as an adjuvant therapy in patients who have undergone RFA alone, in spite of the STORM study indicating that sorafenib is not effective as an adjuvant treatment after ablation or resection (21). In this study, we evaluated the efficacy and safety of sorafenib as an adjuvant treatment for patients who had a complete radiological response after radical treatment by RFA (complete response on imaging).

## Materials and methods

### Patients

The Ethical Committee of the Fifth Medical Center of Chinese PLA General Hospital approved this study, and informed consent was obtained from each patient’s guardian. 683 HCC patients underwent RF ablation treatment at the Fifth Medical Center of the Chinese PLA General Hospital between July 2009 and December 2018. A retrospective analysis of 683 patients was conducted, 164 of whom met the inclusion criteria. The inclusion criteria were as follows: age of 18-75 years; patients who did not receive liver transplantation; HCC patients confirmed by pathology or imaging; HCC patients who did not receive previous treatment except for the RFA or liver resection; diameter of solitary tumor of 5.0 cm or lower; multiple tumors with each having a diameter of 3.0 cm or lower; three or fewer tumors; no invasion into the major portal and hepatic vein branches; no extrahepatic metastases; no severe liver dysfunction (Child-Pugh class C) or no significant coagulopathy (platelet count, <40 × 10^9^/mm^3^; prothrombin activity, <40%); and Eastern Cooperative Oncology Group (ECOG) performance status of 0 and 1. Patients with incomplete clinical data, infection with HIV or other malignancies, or prior anti-cancer treatment for HCC, including sorafenib, were excluded from the study. As a conclusion, 87 patients who underwent RFA alone were compared with 77 patients who underwent RFA+Sor treatment.

### Clinical data collection

Patients’ baseline clinical data were extracted from medical records; they included their age, their sex, their ECOG performance status, their RFA sessions, their laboratory and pathological examinations, their MR and CT images, and their treatment procedures.

### Anesthesia method

Dexmedetomidine hydrochloride (Jiangsu Hengrui Medicine Co., Ltd., Aibeining: 2mL; 200μg) 0.5μg/kg/h was infused intravenously 30 min before RFA, and the infusion was continued for 15 minutes. In order to anesthetize the local area, lidocaine hydrochloride was administered (2%; Northeast Pharmaceutical Group Co., Ltd., Shenyang, China). Additionally, patients were given pethidine hydrochloride (100 mg, Northeast Pharmaceutical Group Co., Ltd., Shenyang, China) according to the actual situation, 25–100 mg at a time.

### Treatment procedures

All patients underwent percutaneous radiofrequency ablation under dual-phase spiral CT guidance (GE Medical Systems, Milwaukee, WI). During the RFA procedure, we used bipolar radiofrequency ablation probes (Celon ProSurge 150-T30 or Celon ProSurge 150-T40, Celon AG Medical Instruments) with active tips of 30-40 mm measuring 1.8 mm in diameter, 1-250 W output power, 470 kHz frequency, and three bipolar channels. Prior to RFA, all patients underwent plain MR imaging and enhanced MR imaging in order to identify the number, location, and scope of lesions. A CT scan was used for localization during RFA treatment, and the tumor center was selected for puncture as a result of a preoperative MRI examination. Following probe insertion, a CT scan was performed, and RFA was performed once an accurate localization had been achieved. When a non-enhancing region was greater than the previous tumor area, we considered that the RFA treatment was complete. Multiple overlaps were abated using the technique of Chen et al. (22), if the largest tumor diameter exceeded 3 cm.

Sorafenib (Bayer Pharma, Germany) treatment was initiated at the time point of 3 days post-radiofrequency ablation, once liver function had returned to near-normal levels. All patients achieved a complete response at the imaging level following RFA, with the assessment conducted immediately post-RFA. Each patient received a standard dose of sorafenib (400 mg, once daily, orally). If drug-related adverse events occurred, therapy was interrupted or dose reduced (200 mg once daily, orally). Symptomatic treatment was provided to patients who experienced adverse reactions to the medication. Whenever a grade 3 adverse event occurred, treatment interruption was recommended. It is also recommended to discontinue sorafenib treatment when tumor recurrence is observed in the patient.

### Follow-up assessments

Each patient was examined at baseline and their medical histories were recorded. Child-Pugh class, ECOG performance status, alpha fetal protein (AFP) levels, and routine laboratory tests were conducted. CT and MR imaging were used to assess tumors. Patients in the RFA+Sor group were recorded as to their sorafenib medication status. One month after RFA, all patients underwent abdominal contrast-enhanced CT or MR imaging, as well as routine laboratory testing. Every three months, the treatment response was assessed. After two to five years after RFA, routine examinations were extended to every four months, and after five years to every six months. The MR and CT images were evaluated independently by two authors with more than ten years of experience in a blinded manner.

When CT was unable to confirm the presence of a residual tumor, MR imaging was performed. All CT examinations, including plain and contrast-enhanced exams, were conducted using a multi-detector row CT scanner (GE Medical Systems, Milwaukee, WI, USA) for the sequential acquisition of 8-mm thick sections at 120 kV and 300 mA. At a flow rate of 3.0-4.5 mL/s, patients were administered intravenous non-ionic contrast agents (iopromide injection; iodine concentration 1.5-2 mL/kg, 300-350 mg I/mL; Bayer Pharma, Berlin, Germany). Following the injection of contrast agents, arterial phase, venous phase, and delayed phase scans were obtained after 30, 60, and 120 seconds.

A 1.5-T MR unit (GE Medical Systems, Milwaukee, WI, USA) was used to perform MR imaging with a 16-channel phased-array body coil. Non-enhanced T1-weighted gradient-echo (TR/TE: 210/2.3 ms; 80°flip angle; 256 × 134 matrix; number of excitations = 1; 8-mm thick section with 1-mm spacing) and respiratory-triggered T2-weighted fast spin-echo (TR/TE: 1900/64 ms; 128 × 128 matrix; number of excitations = 1; 8-mm thick section with 1-mm spacing) pulse sequences were obtained. A pre-saturation band was applied above and below the scanning field to eliminate the influence of lung gas, heartbeats, intestinal gas, and gastrointestinal peristalsis on the imagey. Artifacts related to chemical shift were eliminated using fat-suppression techniques. The images were acquired during two breath-holds. The same technical parameters were used for the dynamic enhanced scans following plain scanning. The dynamic contrast-enhanced MRI examination was performed using gadobenate dimeglumine (0.15 mmol/kg, MultiHance; Bracco Sine, Shanghai, China) at a flow rate of 3 mL/s. Arterial phase, venous phase, equilibrium phase, and delayed phase scans were performed 20, 50, 90, and 600 seconds after injection of contrast agents.

The overall survival (OS) was defined as the period of time from the start of RFA treatment to the date of death or loss of follow-up. The recurrence-free survival (RFS) was defined as the period between the start of RFA treatment and the occurrence of a recurrence of the liver tumor, the progression of lymph node metastases, or the development of distant metastases. During hospitalization, all adverse events were evaluated and documented by telephone follow-up after discharge. CTCAE v5.0 was used to record and assess their performance.

Tumor progression in the treatment area after ablation was defined as tumor enhancement. A distant recurrence refers to the appearance of a new tumor in an untreated area of the liver or in an extrahepatic region (23). For local tumor progression or recurrence, treatment options included RFA, transcatheter arterial chemoembolization (TACE), chemotherapy, or conservative treatment, based on tumor status, routine laboratory tests, results of the multidisciplinary team’s discussion, and patient preference.

### Statistical analysis

The statistical analyses were conducted using SPSS version 24.0 (International Business Machines Corp., USA) and GraphPad Prism version 8.0 (GraphPad, USA). The data are presented as counts (%) and means with standard deviations. Based on independent assessment, a subgroup analysis of OS was performed using Cox regression. Survival curves were generated using the Kaplan-Meier method, and the data were compared using the log-rank test with two-sided P-values. The baseline characteristics and safety profiles of the two treatment groups were compared using the Chi-squared test and the t-test. P < 0.05 was considered statistically significant.

## Results

### Baseline patient data

**Figure 1:**
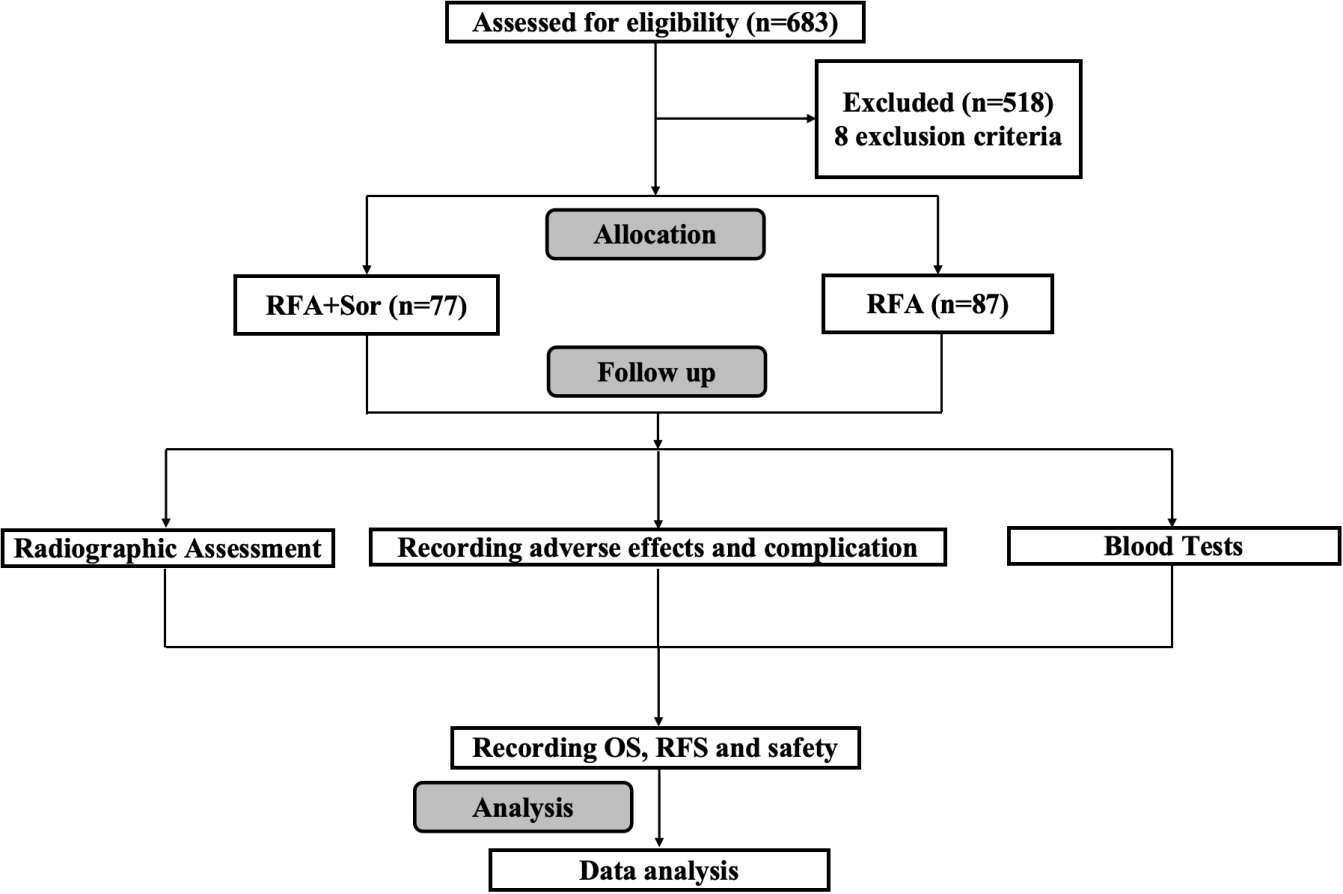
Flowchart of the study profile.

A total of 683 patients were reviewed, but only 164 were included in the study. One group received RFA treatment (n=87) and the other received sorafenib adjuvant treatment after RFA (RFA+Sor, n=77). As shown in **Figure 1**, the study process is summarized.

The baseline characteristics of the two groups were balanced, and none of the compared characteristics showed significant differences between the two groups (*P*>0.05). As shown in **Table 1**, the baseline characteristics of the population are described. The mean ages of the RFA group and the RFA+Sor group were 56.6 ± 8.6 and 56.1 ± 9.6 years, respectively. In the RFA group, 68 patients (77.3%) were males, while in the RFA+Sor group, 59 patients (76.6%) were males. In both RFA and RFA+Sor groups, hepatitis B virus infection was the main cause of HCC. Most of the patients had a good physical condition, an ECOG performance status of zero, and a preserved liver function. After tumor recurrence, there was no statistically significant difference in the number of RFA sessions between the RFA and RFA+Sor groups.

**Table 1:** Baseline characteristics of the study patients (n=165).

Parameter	RFA+Sor (n=77)	RFA (n=87)	*P*-Value
Age	56.1±9.6	56.6±8.6	0.693
Sex			0.921
M	59(76.6)	68(77.3)	
F	18(23.4)	20(22.7)	
ECOG performance status			0.549
0	50(64.9)	61(69.3)	
1	27(35.1)	27(30.7)	
No. of tumours			0.070
1	27(35.1)	45(51.1)	
2	11(14.3)	6(6.8)	
3	39(50.6)	37(42.1)	
Tumor size			0.114
≤ 3 cm	55(71.4)	72(81.8)	
3.1-5.0 cm	22(28.6)	16(18.2)	
BCLC stage			0.298
A	38(49.4)	50(57.5)	
B	39(50.6)	37(42.5)	
Hepatitis B surface antigen			0.152
Positive	61(79.2)	77(87.5)	
Negative	16(20.8)	11(12.5)	
AFP level (ng/ml)	325.9±933.7	325.8±1418.5	1.000
Serum albumin level (g/L)	36.8±5.7	36.8±5.4	0.876
Serum alanine aminotransferase level (U/L)	37.2±35.6	38.9±30.6	0.759
Serum glutamic aminotransferase levels (U/L)	51.1±47.4	43.6±31.1	0.235
Total bilirubin level (umol/L)	18.0±10.4	21.2±33.1	0.435
RFA sessions			0.415
≤ 1	17(22.1)	15(17.0)	
> 2	60(77.9)	73(83.0)	

Data were presented as count and percentage.

ECOG, Eastern Cooperative Oncology Group.

### Survival and disease recurrence

**Figure 2:**
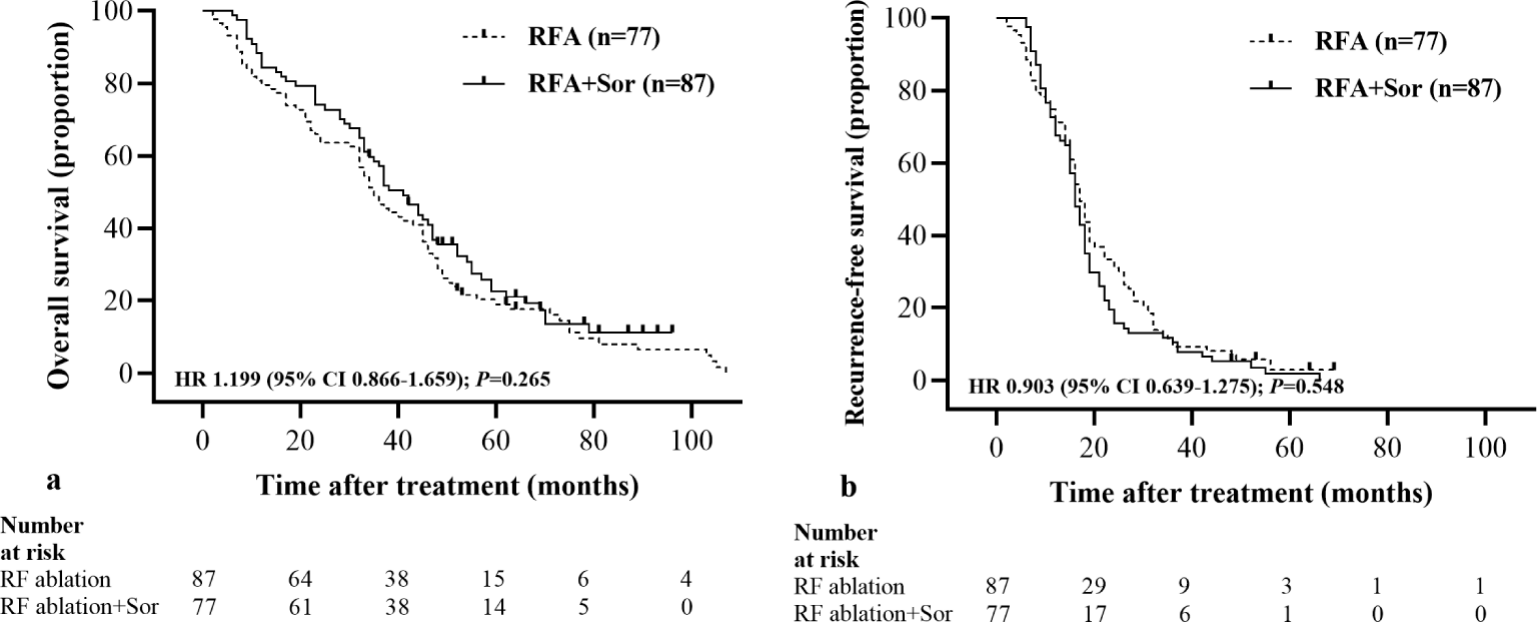
Kaplan-Meier curves show all participants’ OS and RFS. **(A)** median OS is 35.0 months (95% CI: 29.99, 40.01) in the RFA group and 41.0 months (95% CI: 34.28, 47.72) in the RFA+Sor group; **(B)** median RFS is 12.0 months (95% CI: 5.69, 18.31) in the RFA group and 15.0 months (95% CI: 7.03, 22.97) in the RFA+Sor group. HR=hazard ratio.

The median OS in the RFA group was 35.0 months (95% CI: 29.99, 40.01) and 41.0 months (95% CI: 34.28, 47.72) in the RFA+Sor group (HR, 1.199; 95% CI: 0.866, 1.659, *P*=0.265) (**Figure 2A**).

The median RFS for the RFA group was 17.0 months (95% CI: 15.17, 18.83) and 16.0 months (95% CI: 14.44, 17.56) for the RFA+Sor group (hazard ratio [HR], 0.845; 95% CI: 0.618, 1.156, *P*=0.267; **Figure 2B**). In **Table 2**, treatment methods for recurrent HCC following RFA are shown, and there was no statistically significant difference between the two groups.

**Table 2:** Treatment for Recurrence.

Treatment	RFA+Sor (n=69)	RFA (n=64)	*P*-Value
RF ablation	36(52.2)	31(48.4)	0.667
TACE	47(68.1)	35(54.7)	0.112
Systemic chemotherapy	0(0)	0(0)	…
Conservative treatment	6(8.7)	7(10.9)	0.664

Data were presented as count and percentage.

Data are numbers of recurrence patients.

**Figure 3:**
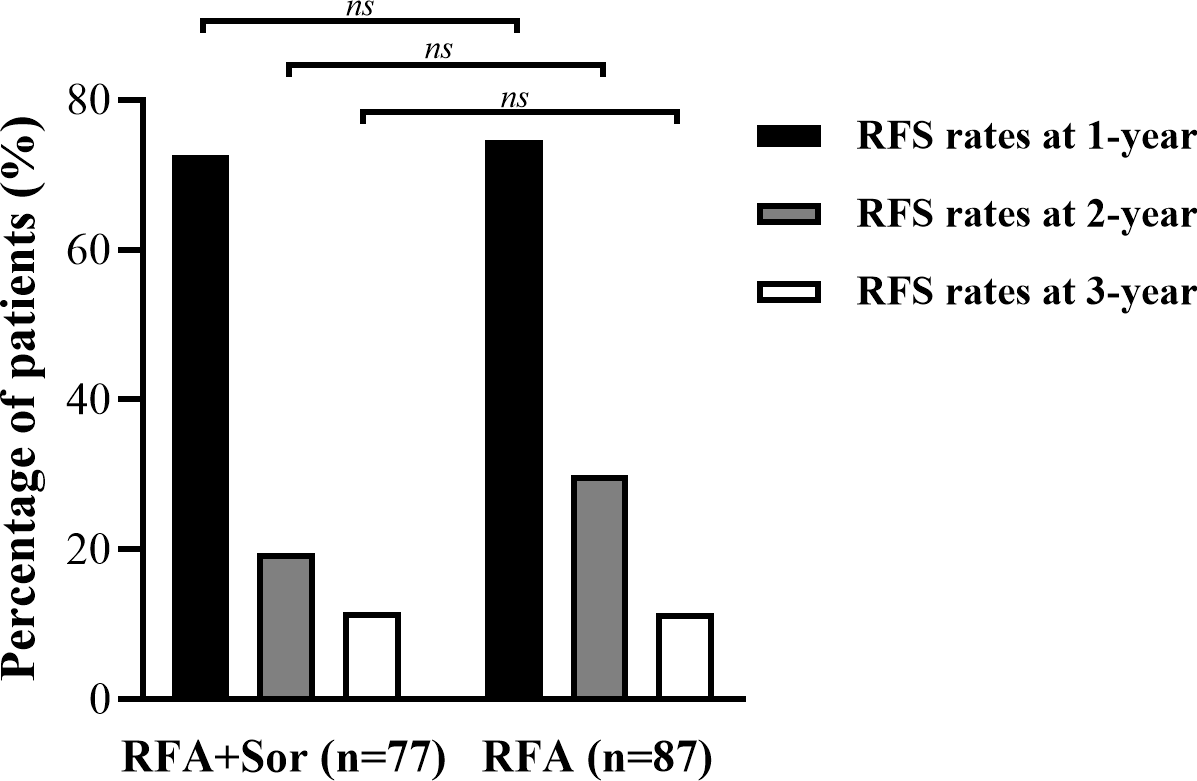
Bar graph shows the RFS of 1-year, 2-year and 3-year in RFA group are 74.7%, 29.9%, and 11.5% and the RFS of 1-year, 2-year and 3-year were 72.7%, 19.5%, and 11.7% in RFA+Sor group. ns, Not Significant.

**Figure 4:**
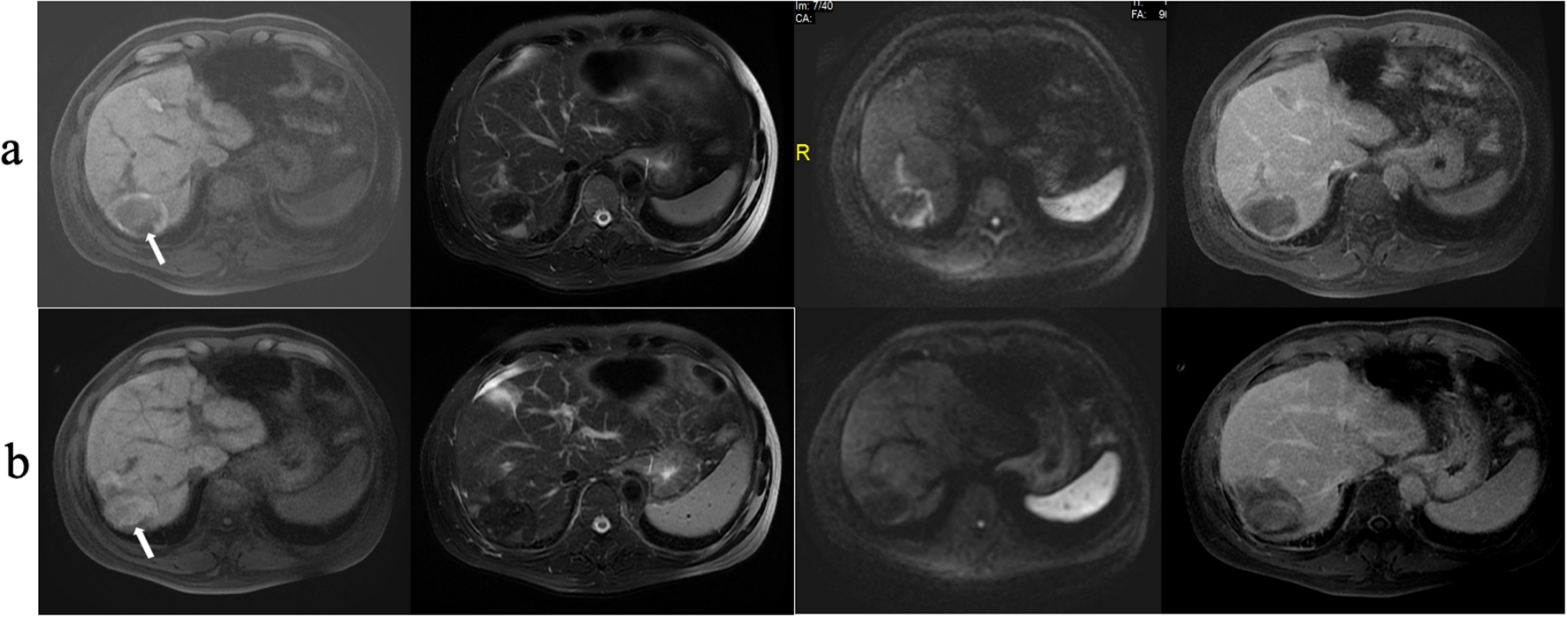
RFA treatment of HCC. **(A)** MRI shows a HCC lesion 4.9 cm in diameter; **(B)** MRI scanning at 12 months after RFA treatment shows that the ablated area had shrunk significantly.

For the RFA group, the 1-, 2-, and 3-year RFS rates were 74.7%, 29.9%, and 11.5%, respectively. The 1-, 2-, and 3-year OS for the RFA+Sor group were 72.7%, 19.5%, and 11.7%, respectively. There was no statistically significant difference between 1-, 2- and 3-year RFS (**Figure 3**). Representative images of tumor after RFA treatment are shown in **Figures 4A, B**.

### Subgroups analysis

**Figure 5:**
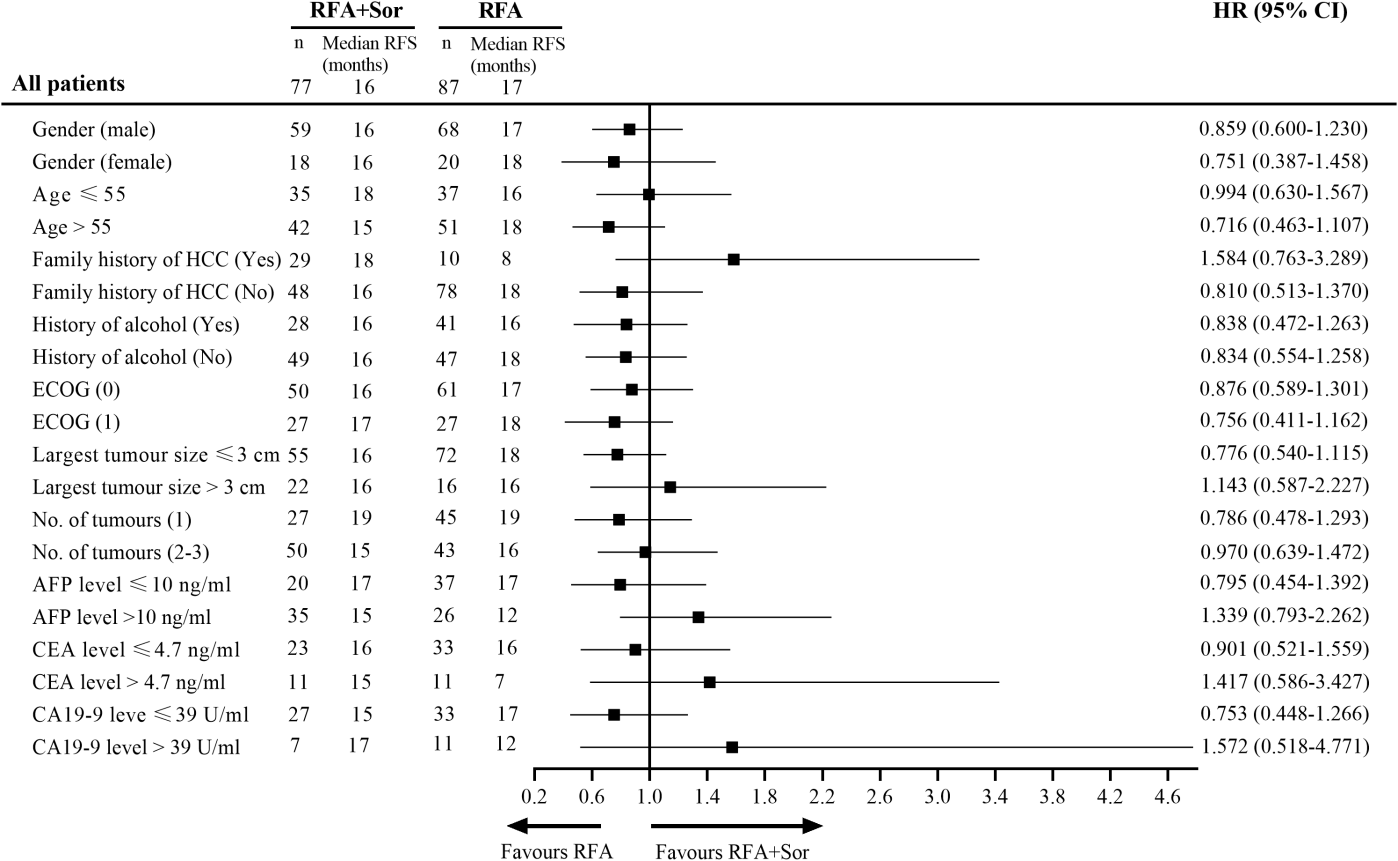
Subgroup analysis of RFS by Cox regression based on independent assessment. RFS, recurrence-free survival; HR, hazard ratio; ECOG, Eastern Cooperative Oncology Group; AFP, alpha fetal protein; CEA, carcino-embryonic antigen; CA, cancer antigen.

Cox regression analysis was used to analyze the information of 164 patients with HCC (**Figure 5**). A statistically significant difference was not observed among the subgroups defined by baseline stratification factors, including gender, age, family history of HCC, history of alcohol abuse, Eastern Cooperative Oncology Group performance status, number of tumors, and tumor marker. The difference in RFS between the RFA group and the RFA+Sor group was not statistically significant (*P*>0.05) for all subgroups.

### Medication status of sorafenib

The median duration of sorafenib treatment in the RFA+SOR group was 10.0 months (95% CI: 9.22-10.78). In total, 77.92% (60/77) of patients receiving sorafenib required a dose reduction, and 85.71% (66/70) required its discontinuation (**Table 3**).

**Table 3:** Sorafenib exposure.

Treatment	Duration of treatment (months)	Dose reduction	Dose interruption
RF ablation+Sor (n=77)	10 (9.22-10.78)	60 (77.92)	66 (85.71)

Data were presented as count and percentage.

In terms of reasons for discontinuation, disease recurrence was the most common reason (81.82% [54/66] patients); 15.15% (10/66) of patients discontinued sorafenib due to adverse events; and 3.03% (2/66) of patients died (**Table 4**).

**Table 4:** Reasons for discontinuation.

Treatment	Disease recurrence	Adverse events	Death
RF ablation+Sor (n=66)	54 (81.82)	10 (15.15)	2 (3.03)

Data were presented as count and percentage.

### Safety analysis

We analyzed the safety data of 164 patients (**Table 5**). There were no treatment-related deaths in our study, and all complications were grade III. A majority of the adverse events related to RFA were diarrhea (RFA group, 16/87 [18.4%] vs. RFA+Sor group, 23/77 [29.9%], P=0.078), vomiting (RFA group, 19/87 [21.8%] vs. RFA+Sor group, 18/77 [23.4%], P=0.930), abdominal pain (RFA group, 23/87 [26.4%] vs. RFA+Sor group, 20/77 [18.2%]), fatigue (RFA group, 18/87 [20.7%] vs. RFA+Sor group, 14/77 [18.2%], P=0.863), and hypertension (RFA group, 22/87 [25.3%] vs. RFA+Sor group, 17/77 [22.1%], P=0.797). In the RFA+Sor group, 15/77 (19.5%) patients developed skin rashes, while in the RFA alone group, none of the patients developed skin rashes.

**Table 5:** Common toxic effects encountered after treatment.

Toxic Effect	RFA+Sor (n=77)	RFA (n=87)	*P*-Value	Grade
Skin rashes	15 (19.5)	0 (0)	0.001	I-II
Diarrhea	23 (29.9)	16 (18.2)	0.078	I-II
Vomiting	18 (23.4)	19 (21.6)	0.930	I-II
Abdominal Pain	20 (26.0)	23 (26.1)	0.981	I-II
Fatigue	14 (18.2)	18 (20.5)	0.863	I-II
Hypertension	17 (22.1)	22 (25.0)	0.797	I-II
Death	0 (0)	0 (0)	…	I-II

Data were presented as count and percentage.

The description was based on 164 patients records.

## Discussion

A retrospective study of sorafenib as an adjuvant treatment after RFA for early HCC found no difference in OS or RFS between the two groups. Thus, this study did not demonstrate that sorafenib is an excellent adjuvant treatment in these patients.

A major reason for treatment failure after radical treatments such as liver resection and local ablation is the high rate of recurrence in patients with HCC. Several studies have reported 5-year recurrence rates and median survival rates for early HCC patients of 50-70% and 30-70 months, respectively (5–9, 24–28). It is urgently necessary to use adjuvant treatment in order to prevent the recurrence of HCC and prolong survival.

In our retrospective study, we found that the OS and RFS of patients treated with sorafenib as adjuvant treatment following RFA were similar to those treated with RFA; Likewise, comparing the RFA group to the RFA+Sor group, sorafenib as an adjuvant treatment did not demonstrate any advantages in any subgroups. The potential reasons for the ineffectiveness of sorafenib as adjuvant therapy after RFA are as follows: First, sorafenib has primarily demonstrated efficacy in the palliative treatment of HCC and is not the first choice for adjuvant therapy. Additionally, sorafenib is a relatively weak antitumor drug with only marginal benefits. Therefore, its mechanisms of inhibiting tumor angiogenesis and cell proliferation may be insufficient to prevent minimal residual disease from progressing to recurrence after local treatments like RFA. With advances in oncology, more promising agents have emerged, and sorafenib is no longer the preferred drug. Agents such as lenvatinib and regorafenib may serve as alternative options for adjuvant therapy following RFA.

Unfortunately, 85.71% of patients had to discontinue using sorafenib for a variety of reasons due to disease recurrence (81.82%). The multiple mechanisms of drug resistance of sorafenib, which could be mediated by a variety of signaling pathways (29, 30), lead to tumor recurrence, which makes it difficult for physicians and patients to accept the continued use of sorafenib. Adjuvant sorafenib treatment after RFA also increases the financial burden on patients. As a result of sorafenib resistance, we will need to explore the selection of TKIs in the future.

There were no treatment-related deaths in this study. It has been demonstrated in previous studies that sorafenib alone and TACE plus sorafenib are safe and effective in the treatment of advanced HCC (18, 31, 32). According to our study, skin rashes and diarrhea were the most common adverse reactions, which is similar to the findings of Llovet et al. (18). In the RFA+Sor group, there was a higher incidence of skin rashes and diarrhea than in the RFA group, and the main cause was oral sorafenib. Our study, however, reported a lower incidence of HCC than previous studies (33, 34), since the patients in this study had early HCC and their physical condition was better than that of patients with advanced HCC. In addition, the median duration of sorafenib treatment was shorter than in previous studies, and 85.71 percent of patients had their dose interrupted. According to this study, physicians and patients have difficulty accepting complications, and patients prefer surgical procedures, including liver resections or local ablations, over oral medications. Other adverse effects, such as vomiting, abdominal pain, and hypertension, are common, and this study’s findings are similar to those of previous studies (25, 35).

There are some limitations to this study. In this retrospective study, it was not possible to predict whether the patients would take sorafenib or not. Several clinical factors, including the number of tumors and the differentiation of tumor cells, as well as the economic situation may influence the use of sorafenib in these patients, making them different from others who received sorafenib treatment. Additionally, we carefully balanced some clinical characteristics of the patients, but confounding factors were inevitable in this study; other treatments, the size and number of tumors, and their combinations may influence the outcome to some extent. This issue will be addressed in a prospective study. The number of HCC patients who underwent other tumor marker examinations, such as carcinoembryonic antigen and cancer antigen 19-9, was limited, in addition to the AFP level. Therefore, it may be difficult to identify significant differences between these subgroups. In the future, a prospective study will be initiated, and all patients receiving adjuvant sorafenib treatment will undergo a complete laboratory examination.

As a result, we conclude that tumor recurrence rates among patients treated with RFA+Sor were not higher than those treated with only RFA. Therefore, RFA+Sor is not considered to be an effective treatment for patients with HCC. It will be necessary to conduct prospective randomized controlled trials in the future in order to confirm these findings.

## Data Availability

The raw data supporting the conclusions of this article will be made available by the authors, without undue reservation.
